# Transcriptomic Profile of Perineural Invasion in Prostate Cancer Identifies Prognostic Gene Signatures

**DOI:** 10.3390/biomedicines13081789

**Published:** 2025-07-22

**Authors:** Cagdas Aktan, Swati Mamidanna, Caryn Cobb, Ceren Atalar, Jacqueline Chan, Christina M. Breneman, Okan Argun, Mutlay Sayan

**Affiliations:** 1 Department of Medical Biology, Faculty of Medicine, Bandirma Onyedi, Eylul University, Balikesir 10250, Türkiye; 2 Department of Radiation Oncology, Rutgers Cancer Institute of New Jersey, Rutgers Robert Wood Johnson Medical School, New Brunswick, NJ 08901, USA; 3 Department of Radiation Oncology, Brigham and Women’s Hospital and Dana-Farber Cancer Institute, Harvard Medical School, Boston, MA 02115, USA

**Keywords:** prostate cancer, perineural invasion, prognostic biomarkers

## Abstract

**Background:** Prostate cancer is a common malignancy among men worldwide, with various histopathologic features that influence its progression and prognosis. One such feature is perineural invasion (PNI), which has been associated with aggressive disease. In this retrospective study, we analyzed genomic alterations associated with PNI in patients who underwent radical prostatectomy. **Methods:** A total of 421 prostate cancer patients who underwent radical prostatectomy without neoadjuvant therapy were identified from The Cancer Genome Atlas. PNI was present in 378 patients (89.8%) and absent in 43 (10.2%). Differentially expressed genes were identified, and mRNA expression levels of key genes were analyzed. The prognostic significance of these genes was evaluated using log-rank tests and Cox proportional hazards models to estimate hazard ratios and 95% confidence intervals. **Results:** Levels of *COL9A3*, *ASPN*, *ESR1*, *MUC1*, *PIP*, *SFRP4*, *KRT19*, *CLDN1*, and *COMP* were significantly higher in the tumor tissues of patients in the PNI group compared to those in the non-PNI group (*q* < 0.05), and *RYR2*, *MME*, and *AZGP1* expression levels were significantly higher in the non-PNI group (*q* < 0.05). A high mRNA expression level of *AZGP1* was associated with longer disease-free survival, whereas high mRNA expressions of *ASPN*, *COMP*, *RYR2*, and *SFRP4* were associated with shorter disease-free survival. **Conclusions:** Prostate cancer patients with genomic alterations associated with PNI may face a higher risk of disease progression after prostatectomy, highlighting the need for further prospective studies to validate these findings.

## 1. Introduction

Prostate cancer is among the most commonly diagnosed malignancies in men and exhibits a wide spectrum of clinical behavior from indolent tumors that may not require immediate treatment to aggressive cancers necessitating prompt intervention [1,2]. This variability highlights the importance of precise risk stratification to guide treatment decisions and improve patient outcomes. Recent research efforts have focused on identifying novel biomarkers that enhance prognostication and refine therapeutic strategies [3,4].

Perineural invasion (PNI), defined as the presence of cancer cells tracking along or around nerve fibers, is one such histopathologic feature of interest. PNI has been associated with adverse pathological outcomes, including an increased risk of extraprostatic extension and higher Gleason grade on biopsy [5,6,7]. In the post-prostatectomy setting, PNI has also been linked to a higher incidence of biochemical recurrence, suggesting its potential role as an independent prognostic factor [8,9,10]. Molecular studies further support this association, demonstrating increased proliferation and upregulation of growth-promoting genes within tumor foci exhibiting PNI [11]. These findings raise the hypothesis that PNI may not simply be a marker of aggressive disease but may actively contribute to a pro-tumorigenic microenvironment.

While histologic associations with PNI have been extensively described, its underlying genomic characteristics are not well defined. In this study, we utilized data from The Cancer Genome Atlas (TCGA) to examine the molecular profile of prostate tumors with and without histopathologic evidence of PNI. Our goal was to identify differentially expressed genes and genomic alterations associated with PNI and to evaluate their prognostic significance in patients undergoing radical prostatectomy.

## 2. Materials and Methods

### 2.1. Study Population

In this retrospective study, we identified patients with a diagnosis of prostate cancer who underwent upfront radical prostatectomy and had no prior neoadjuvant therapy, using data from TCGA. Clinical, genomic, and pathologic data were extracted from the TCGA database [12]. PNI status was determined based on information reported in the original pathology reports provided within the TCGA dataset, and patients were categorized into PNI and non-PNI groups accordingly for subsequent molecular and prognostic analyses. Although pathology reports across contributing institutions followed general guidelines, the exact criteria used to define PNI may have varied slightly between centers. Therefore, while the PNI annotation in TCGA is centrally curated, some degree of inter-institutional variability cannot be excluded.

### 2.2. Gene Expression Analysis

The TCGA provides gene expression profiling data generated by high-throughput next-generation sequencing (NGS) platforms, which extract RNA and sequence the cDNA from both fresh-frozen and formalin-fixed paraffin-embedded (FFPE) tissues. Utilizing the RNASeq V2 data, gene-level expression estimates were generated and normalized using RNA-Seq by Expectation-Maximization (RSEM).

Differential expression analysis was conducted using the cBioPortal platform (https://www.cbioportal.org/) (accessed on 20 February 2025) based on log2-transformed RSEM-normalized expression values. Fold-change was calculated as the log2 of the ratio of the (unlogged) mean expression levels between patients with and without PNI. Statistical significance was assessed using a two-tailed Student’s *t*-test, and multiple testing correction was performed using the Benjamini–Hochberg procedure to obtain false discovery rate (FDR)-adjusted q-values. FDR was preferred over Bonferroni correction due to its greater statistical power in large-scale gene expression data and its widespread use in transcriptomic analyses. Genes with an absolute log2 fold change (|log_2_FC|) greater than 0.585 (corresponding to a 1.5-fold change in expression) and a q-value below 0.05 were defined as differentially expressed genes (DEGs). Although formal sensitivity testing with alternative thresholds was not performed, the selected cut-off is supported by prior studies and provides a balance between biological relevance and statistical detectability. This fold-change threshold is commonly used in transcriptomic studies to balance biological relevance with statistical stringency [13].

### 2.3. Construction of the Protein-Protein Interaction Network and Identification of Hub Genes

The identified DEGs were input into the Search Tool for the Retrieval of Interacting Genes (STRING v12.0) to construct a protein–protein interaction (PPI) network [14]. The constructed PPI network was visualized using the Cytoscape software v3.10.3 [15]. To identify hub genes, two Cytoscape plug-ins, Molecular complex Detection (MCODE) v2.0.3 and CytoHubba v0.1, were used to identify significant genes, with parameters set to a K-score of 3, a degree cutoff of 2, a node cutoff of 0.3, and a maximum depth of 100 [16]. Gene clusters identified as significant by both MCODE and CytoHubba were cross-referenced, and the intersecting genes were selected as the final key candidates [17].

### 2.4. Functional Enrichment Analysis

Functional enrichment analysis of the top-ranked hub genes was performed using Gene Ontology (GO) analysis to evaluate their biological significance. GO annotations were used to classify the genes into biological processes, molecular functions, and cellular processes. To further explore the pathways associated with these genes, the profiler web tool (https://biit.cs.ut.ee/gprofiler/gost) (accessed on 20 February 2025) was employed, integrating multiple pathway databases, including the Kyoto Encyclopedia of Genes and Genomes (KEGG), Reactome, and WikiPathways (WP) [18]. This analysis provided insights into the functional roles and regulatory mechanisms of the DEGs.

### 2.5. Genomic Alterations of Genes Compared Between PNI and Non-PNI Groups

From the TCGA database, the genomic alterations dataset was used to further examine differences between the PNI and the non-PNI groups. DNA extraction from FFPE tissues was performed, followed by sequencing using high-throughput NGS technologies [19].

### 2.6. Prognostic Value of Genomic Alterations and Hub Genes

To evaluate whether the genomic alteration of genes or the expression levels of hub genes were significantly associated with patient survival outcomes, such as overall survival (OS) and disease-free survival (DFS), the log-rank test was performed using the GEPIA platform (http://gepia.cancer-pku.cn/) (accessed on 20 February 2025). Cox proportional hazards regression was then used to estimate hazard ratios (HRs) and 95% confidence intervals.

## 3. Results

### 3.1. Study Population and Gene Expression Analysis

A total of 421 patients were identified from the TCGA database who met the eligibility criteria, and of these, 378 patients (89.79%) had PNI on histopathology examination, and 43 (10.21%) were non-PNI. DEGs were demonstrated in 224 of the 19,574 genes assessed.

### 3.2. Hub Genes in the Protein-Protein Interaction Network

**Figure 1:**
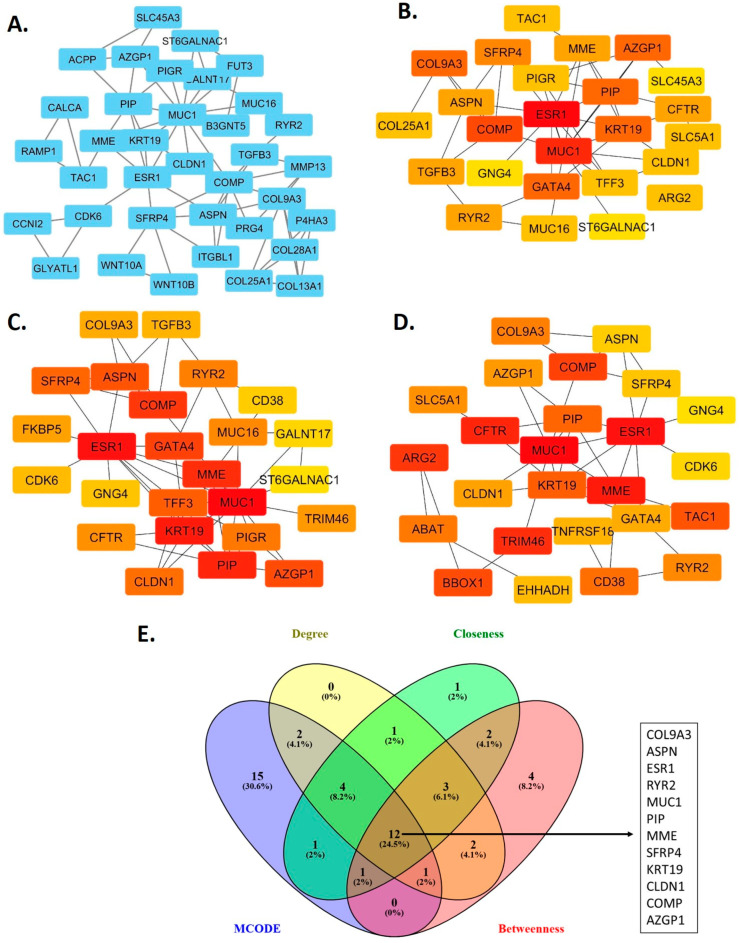
Protein–protein interaction (PPI) network analysis of differentially expressed genes (DEGs) between PNI and non-PNI prostate cancer groups. Each node represents a protein encoded by a DEG, and each edge (lines connecting the nodes) represents a predicted protein–protein interaction. (**A**) The most significant module within the PPI network, identified using the MCODE Cytoscape (v3.10.3). Top-ranked hub genes identified using CytoHubba ranking algorithms: degree (**B**), closeness (**C**), and betweenness (**D**). (**E**) Venn diagram showing the intersection of hub genes identified by MCODE and all three CytoHubba algorithms, yielding 12 overlapping hub genes.

A total of 212 nodes (representing hub gene protein products) and 192 edges (representing interactions between proteins) were identified in the constructed PPI network. A highly significant *p*-value of 1.0 × 10^−16^ was generated through PPI network enrichment analysis, indicating that the network is non-random and that the genes are functionally interconnected. An interaction network was constructed for the DEGs, and the MCODE algorithm, which ranks clusters based on their scores, was applied. The module with the highest score of 4.114 was selected for detailed visualization of these interactions in patients with PNI. As shown in Figure 1A, this module comprises 36 nodes and 72 edges. Using the CytoHubba tool, the top 25 hub genes among the DEGs were identified through algorithms with ranking methods such as degree, closeness, and betweenness, with the results shown in Figure 1B–D. The common intersecting genes from both MCODE and CytoHubba analyses were then merged using a Venn diagram to identify twelve overlapping hub genes (*COL9A3*, *ASPN*, *ESR1*, *RYR2*, *MUC1*, *PIP*, *MME*, *SFRP4*, *KRT19*, *CLDN1*, *COMP*, and *AZGP1*) in DEGs, as shown in Figure 1E.

**Figure 2:**
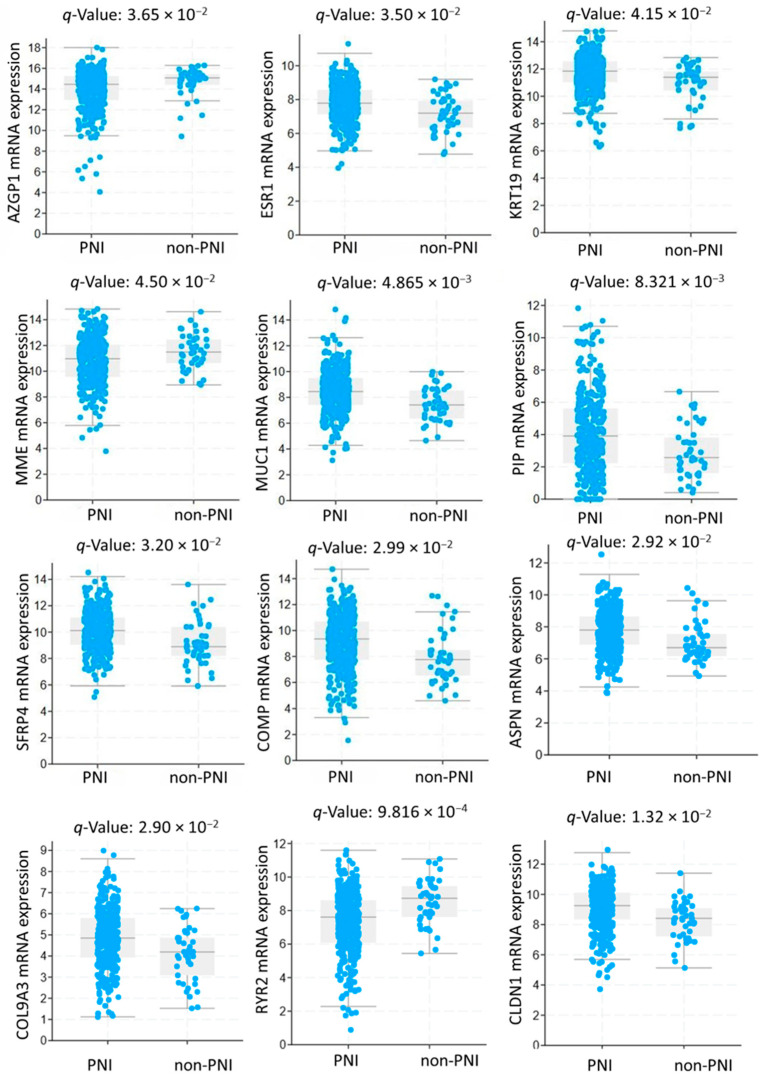
The mRNA expression level of the hub genes. Abbreviations: PNI, perineural invasion. Figure 2 was generated using log2-transformed RSEM expression values from cBioPortal v6.2. Fold-change and statistical comparisons are based on the platform’s calculations using Student’s *t*-test and FDR correction (Benjamini–Hochberg). The plot is intended to illustrate the expression distribution between groups rather than to visually represent raw fold-change magnitude.

An mRNA analysis was done to further compare the expression levels of the hub genes between the two groups. The expression levels of *COL9A3*, *ASPN*, *ESR1*, *MUC1*, *PIP*, *SFRP4*, *KRT19*, *CLDN1*, and *COMP* were significantly higher in tumor tissues of patients with the PNI group compared to those in the non-PNI group (*q* < 0.05), as shown in Figure 2. In the non-PNI group, expression levels of *RYR2*, *MME*, and *AZGP1* were significantly higher (*q* < 0.05) compared to the PNI group.

### 3.3. Genetic Alterations

**Figure 3:**
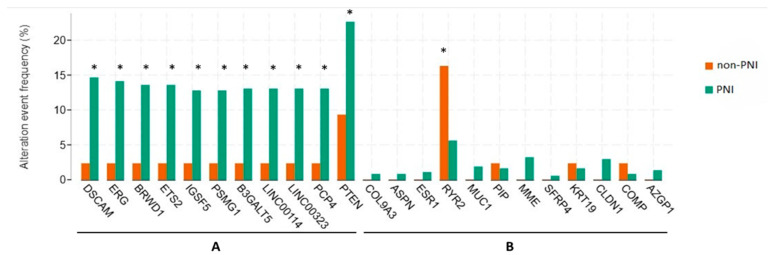
Genetic alterations of genes with the most significant *p*-values (**A**) and the hub genes (**B**). Significance of *p* < 0.05 is marked with a *. Abbreviations: PNI, perineural invasion.

In the PNI group, genetic alterations were significantly observed with increased frequency in the following genes compared to the non-PNI group (*p* < 0.05): *DSCAM* (14.63%, 55 samples), *ERG* (14.10%, 53 samples), *BRWD1* (13.56%, 51 samples), ETS2 (13.56%, 51 samples), IGSF5 (12.77%, 48 samples), PSMG1 (12.77%, 48 samples), B3GALT5 (13.03%, 49 samples), LINC00114 (13.03%, 49 samples), LINC00323 (13.03%, 49 samples), PCP4 (13.03%, 49 samples), and *PTEN* (22.61%, 85 samples), as shown in Figure 3.

### 3.4. Biological Processes and Pathways Underlying Perineural Invasion

Functional enrichment analysis of the top-ranked hub genes revealed significant alterations in several key biological processes relevant to prostate cancer progression in the PNI group. GO biological process analysis identified enrichment in pathways such as estrogen response (GO:0043627), regulation of programmed cell death (GO:0043067), general signaling processes (GO:0023052), nervous system processes (GO:0050877), and the BMP signaling pathway (GO:0030509). These findings suggest that PNI may be associated with transcriptional programs that promote a more invasive or treatment-resistant tumor phenotype.

GO molecular function analysis highlighted critical molecular activities, including estrogen response element binding (GO:0034056), BMP binding (GO:0036122), Wnt-protein binding (GO:0017147), 14-3-3 protein binding (GO:0071889), and p53 binding (GO:0002039). GO cellular component analysis further indicated enrichment in cellular structures such as the extracellular space (GO:0005615), cytoplasm (GO:0005737), and intracellular membrane-bounded organelles (GO:0043231).

Pathway enrichment analysis using KEGG, Reactome, and WP revealed that hub genes were predominantly involved in the Estrogen signaling pathway (KEGG:04915), Endocrine resistance (KEGG:01522), Wnt signaling (KEGG:04310), PI3K-Akt signaling (WP:WP4172), and other cancer-related pathways, including FoxO signaling (KEGG:04068), Renin-angiotensin system (KEGG:04614), Chemical carcinogenesis—receptor activation (KEGG:05207), and innate immune system processes (REAC:R-HSA-168249). These enriched pathways reflect the complex regulatory networks potentially driving PNI-associated disease aggressiveness.

### 3.5. Prognostic Value of Genetic Alteration and Hub Genes

**Figure 4:**
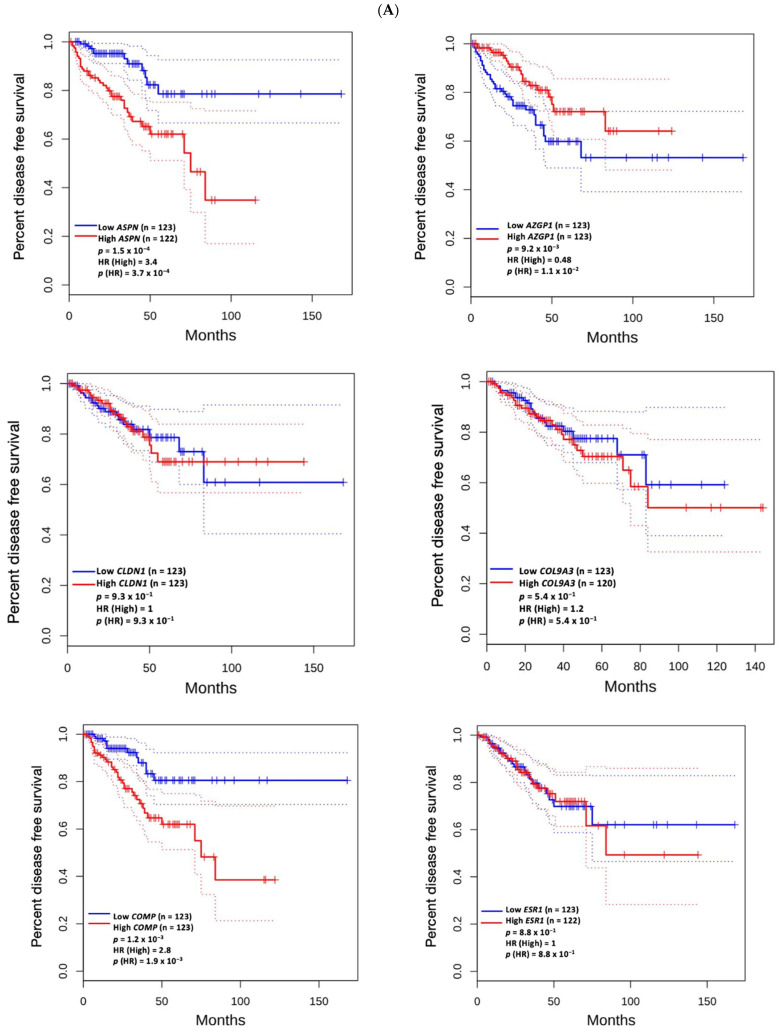

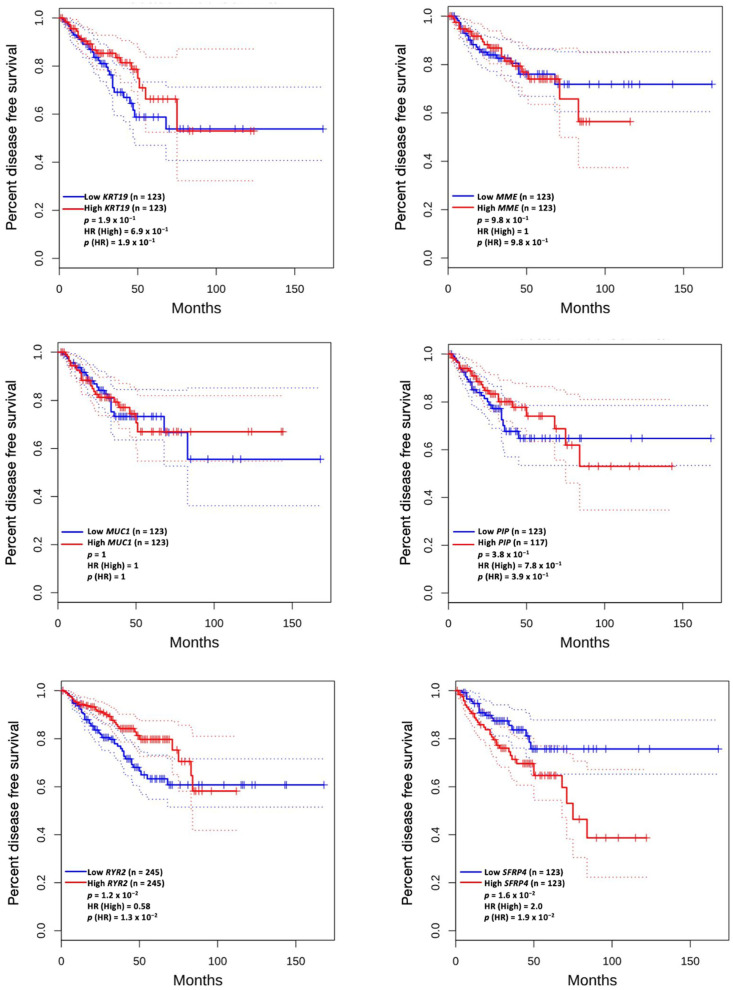

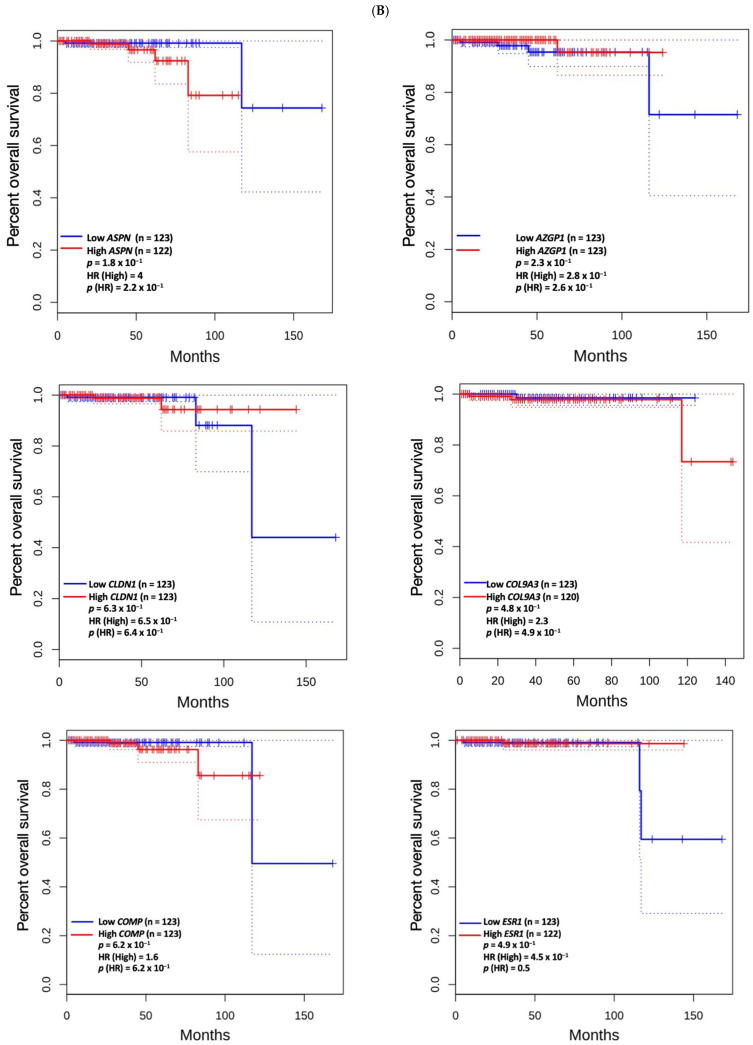

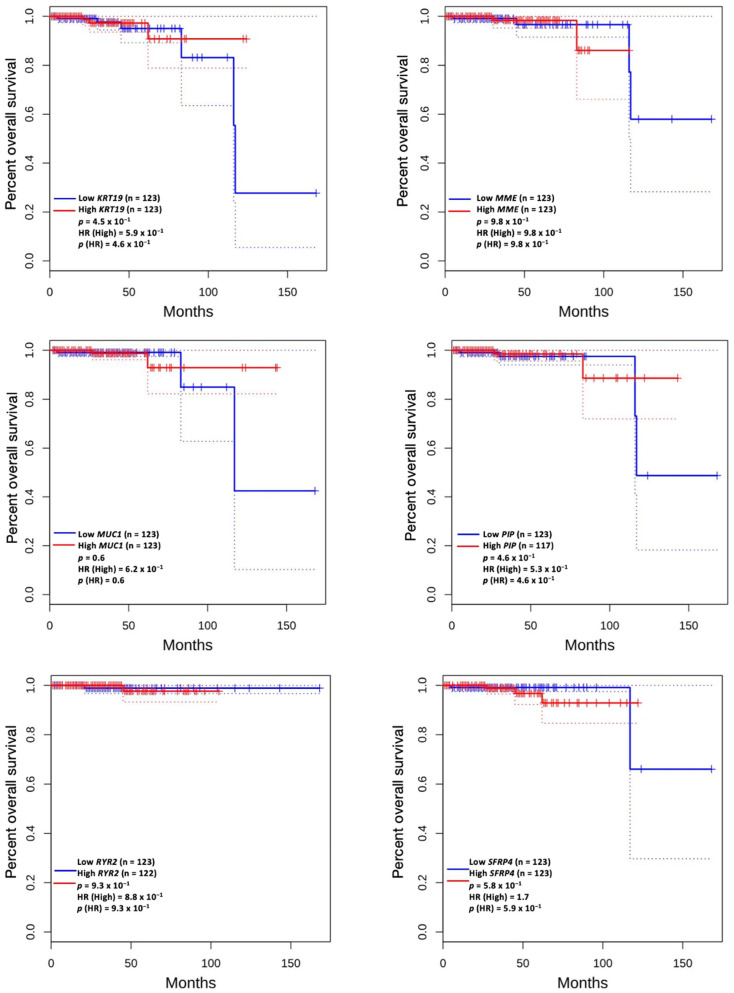
Kaplan–Meier analysis of disease-free survival (**A**) and overall survival (**B**) based on mRNA expression levels of hub genes. Patients were stratified into high- and low-expression cohorts. Red and blue curves represent the high- and low-expression groups, respectively. Solid lines indicate survival estimates; dotted lines represent 95% confidence intervals. The *p*-value reflects the statistical significance of the difference in survival curves between the two groups, as determined by the log-rank test. The hazard ratio (HR) represents the relative risk of an event occurring in the high-expression group compared to the low-expression group, and *p*(HR) indicates the statistical significance of this estimate based on the Cox proportional hazards model. A *p*-value < 0.05 was considered statistically significant for both tests.

Among the identified hub genes, high mRNA expression of *AZGP1* was associated with longer disease-free survival (*p* = 9.2 × 10^−3^). In contrast, elevated expression levels of *ASPN* (*p* = 1.5 × 10^−4^), *COMP* (*p* = 1.2 × 10^−3^), *RYR2* (*p* = 1.2 × 10^−2^), and *SFRP4* (*p* = 1.6 × 10^−2^) were significantly associated with shorter disease-free survival (Figure 4A). No significant association was observed between the expression levels of these hub genes and overall survival (Figure 4B).

**Figure 5:**
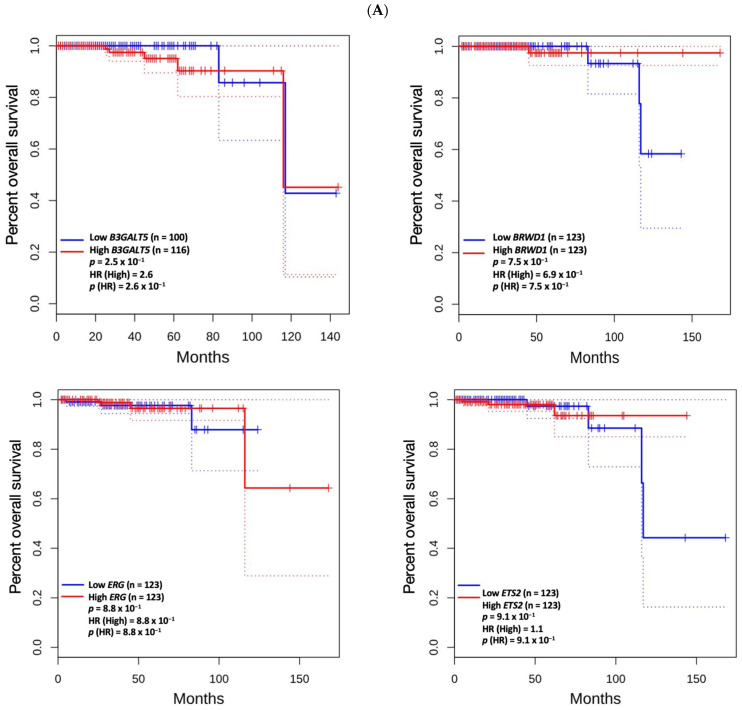

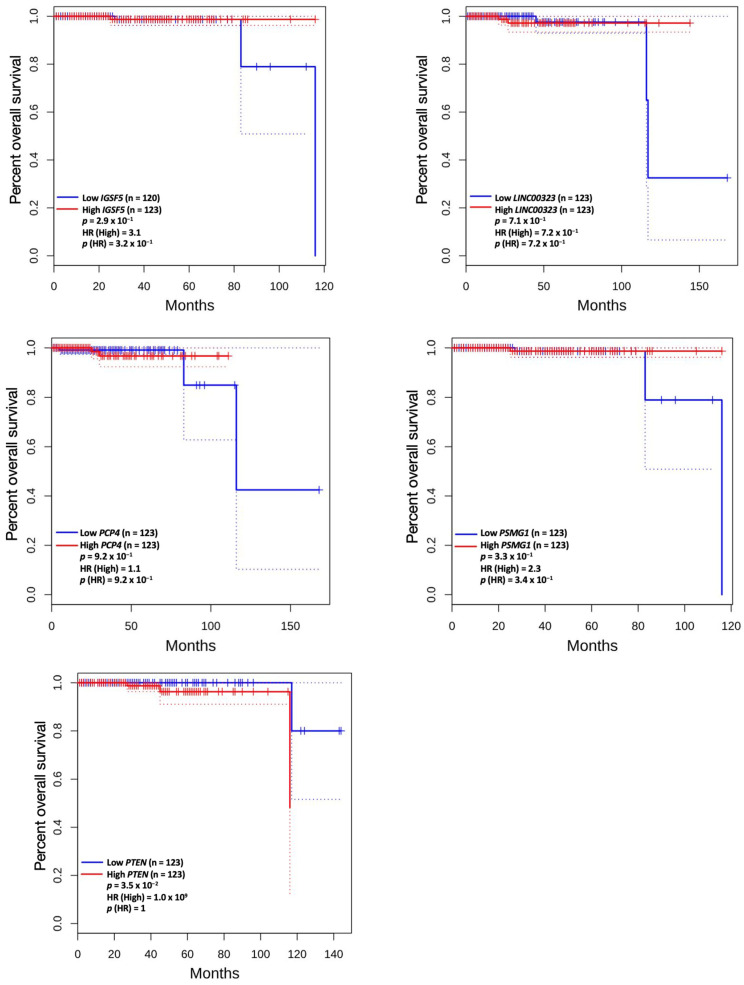

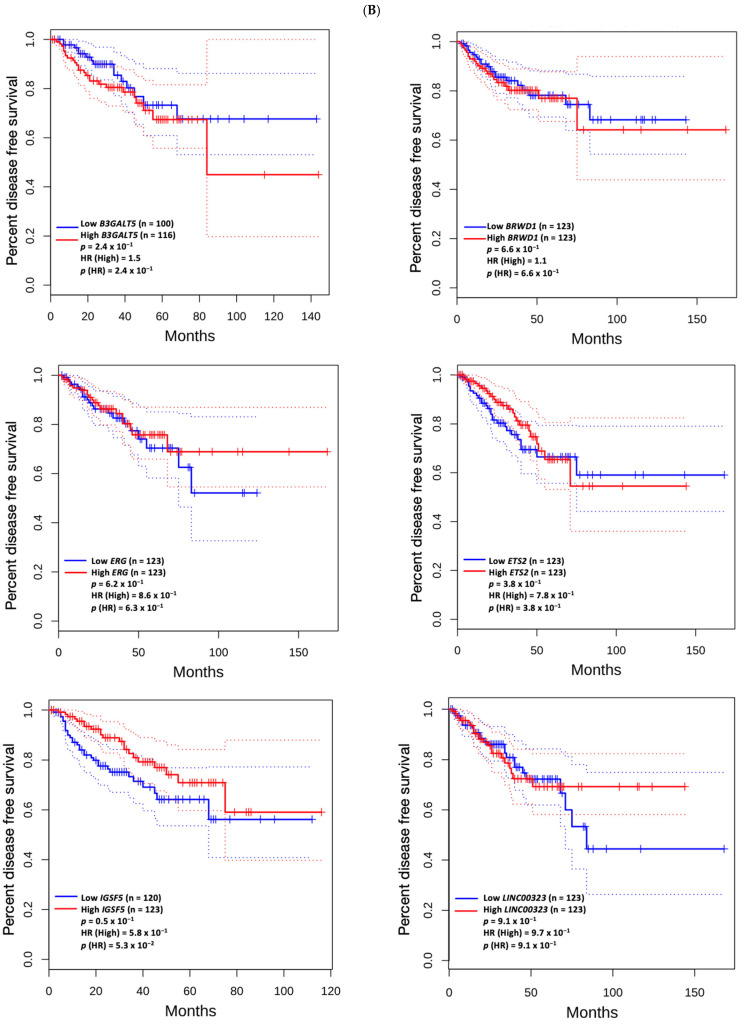

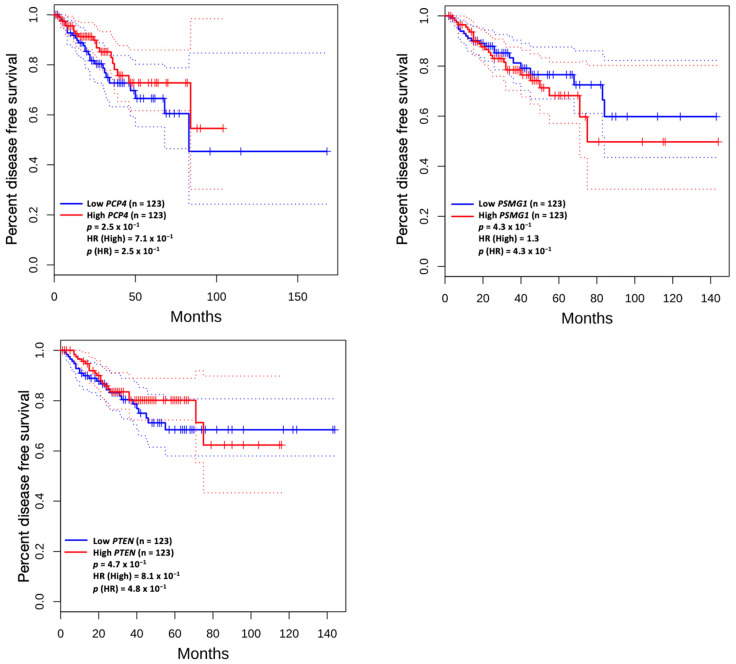
Kaplan–Meier analysis of overall survival (**A**) and disease-free survival (**B**) based on mRNA expression levels of genes with frequent genetic alterations. Patients were stratified into high- and low-expression cohorts. Red and blue curves represent the high- and low-expression groups, respectively. Solid lines indicate survival estimates; dotted lines represent 95% confidence intervals. The *p*-value reflects the statistical significance of the difference in survival curves between the groups, as assessed by the log-rank test. The hazard ratio (HR) represents the relative risk of an event occurring in the high-expression group, and *p*(HR) indicates the significance of this estimate from the Cox proportional hazards model. A *p*-value < 0.05 was considered statistically significant for both tests.

Among the genes with genetic alterations, only *PTEN* showed prognostic significance, with high expression levels associated with shorter overall survival (*p* = 3.5 × 10^−2^; Figure 5A). However, for these genes, no significant associations were found with disease-free survival (Figure 5B).

## 4. Discussion

In this study, we conducted an integrative molecular analysis to characterize the genomic and transcriptional landscape of PNI in prostate cancer. By leveraging gene expression profiling, PPI network construction, genetic alteration analysis, and functional enrichment approaches, we identified molecular features associated with PNI that may underlie its link to aggressive disease behavior. Our network analysis identified 12 hub genes, namely *COL9A3*, *ASPN*, *ESR1*, *RYR2*, *MUC1*, *PIP*, *MME*, *SFRP4*, *KRT19*, *CLDN1*, *COMP*, and *AZGP1* as key molecular players in PNI. Expression profiling revealed that *COL9A3*, *ASPN*, *ESR1*, *MUC1*, *PIP*, *SFRP4*, *KRT19*, *CLDN1*, and *COMP* were upregulated in PNI-positive tumors, while *RYR2*, *MME*, and *AZGP1* were more highly expressed in the non-PNI group. These opposing expression patterns suggest that distinct transcriptional programs may drive tumor phenotypes with or without PNI, potentially influencing invasiveness and prognosis.

While several of these genes have been previously implicated in cancer biology, their specific roles in PNI have yet to be clarified. For instance, although *COL9A3* has been proposed to have tumor-suppressive properties in other malignancies [20,21,22], we hypothesize that its upregulation in PNI-positive tumors may promote perineural spread by facilitating interactions with extracellular matrix (ECM) components such as collagen and proteoglycans [23]. This may enhance tumor cell adhesion and migration along nerve fibers through ECM remodeling. Additionally, *ASPN*, a stromal gene implicated in tumor progression, was also highly expressed in PNI-positive tumors. Prior studies have shown that *ASPN* can support cancer metastasis by modulating TGF-β signaling and ECM organization [24,25,26]. In the context of PNI, *ASPN* may contribute to a permissive microenvironment for neural invasion, potentially through its influence on BMP and Wnt signaling pathways [27].

Our functional enrichment analyses further elucidated the molecular pathways associated with PNI, highlighting alterations in estrogen response, WNT, PI3K-Akt, endocrine resistance, programmed cell death regulation, and nervous system processes. Beyond their biological significance, several of these enriched pathways—such as estrogen signaling, PI3K-Akt signaling, Wnt signaling, and endocrine resistance—are therapeutically actionable and have been linked to treatment resistance in prostate cancer. Notably, cross-regulation between the PI3K-Akt-mTOR and androgen receptor pathways has been shown to contribute to therapeutic resistance, as inhibition of androgen receptor signaling can lead to compensatory activation of the PI3K-Akt pathway through feedback mechanisms [28,29,30]. Similarly, dysregulation of the Wnt signaling pathway has been implicated in the development of castration-resistant prostate cancer and is currently being explored as a therapeutic target [31]. In parallel, increased estrogen receptor signaling has been shown to enhance neural invasion and contribute to a more aggressive disease phenotype, particularly in hormone-refractory prostate cancer [32]. Importantly, the increased expression of *ESR1* in PNI-positive tumors suggests that estrogen signaling might play a crucial role in facilitating nerve invasion [33]. In 1999, *ESR1* was first reported to be associated with metastatic prostate cancer lesions, including those in lymph nodes [34]. Subsequent studies demonstrated that in hormone-refractory prostate cancer, the reactivation of the *ESR1* steroidal pathway could support tumor progression and invasiveness by enhancing the interaction between cancer cells and nerve cells, promoting both local invasion and metastasis [33,35,36]. Taken together, these insights not only enhance our understanding of the molecular underpinnings of PNI but also highlight the potential of targeting these pathways in patients with PNI-positive, high-risk prostate cancer.

We also observed a higher frequency of somatic alterations in several genes—*DSCAM*, *ERG*, *BRWD1*, and *PTEN*—in tumors with PNI. Notably, *PTEN*, a tumor suppressor frequently lost in aggressive prostate cancer, may promote perineural spread via PI3K-Akt pathway activation, enhancing cell survival and invasiveness [37,38,39]. Interestingly, while *RYR2* was more frequently altered in non-PNI tumors and showed higher expression in that group, its elevated expression was paradoxically associated with shorter disease-free survival. These findings suggest that *RYR2* may influence disease progression through mechanisms that differ by PNI status and warrant further investigation.

Several of the hub genes, including *ASPN*, *COMP*, *RYR2*, and *SFRP4*, were associated with shorter disease-free survival, while high *AZGP1* expression correlated with longer survival. These results support the prognostic relevance of these genes and their potential utility in risk stratification. For example, *SFRP4*, a modulator of the Wnt signaling pathway, has been linked to tumor aggressiveness in prostate and other cancers [40,41,42,43,44,45], reinforcing its role as a candidate biomarker.

One notable observation in our study is the considerable prevalence of PNI, which is reported to be 89.8%. This is noticeably higher than the 20–50% range commonly observed in prostatectomy cohorts [46,47]. This increase in prevalence of PNI noted in our study can be explained by the higher-risk profile of patients included in the TCGA-PRAD dataset, which predominantly consists of intermediate- to high-risk prostate cancer cases. PNI is more frequently observed in these high-risk groups, and the rigorous histopathological examination in the TCGA dataset likely leads to more consistent and accurate detection of PNI. These factors may contribute to the observed discrepancy between our findings and those reported in the literature.

Nevertheless, our study has several limitations. First, it is based on retrospective data from the TCGA, and, therefore, further prospective validation in independent cohorts is needed. Second, while we identified associations between gene expression and clinical outcomes, mechanistic studies are required to confirm causality. Specifically, experimental approaches, such as immunohistochemistry, quantitative PCR, or functional assays using prostate cancer and neural cell co-culture systems, could validate the biological relevance of hub genes identified in our study, such as *SFRP4*, *ESR1*, and others, in the context of perineural invasion. Previous studies have provided supporting evidence for the involvement of these hub genes in prostate cancer progression [24,42,48,49,50,51,52,53], which indirectly supports our findings. Nonetheless, dedicated experiments are required to clarify their specific roles in neural invasion. Integration of additional molecular layers, including epigenetic, proteomic, and spatial transcriptomic data, may aid in shedding light on the pathways driving PNI. A further limitation of our study is the marked imbalance in the number of patients in the PNI group (*n* = 378) compared to the group without PNI (*n* = 43). This discrepancy was not due to sample selection bias but rather a reflection of the inherent distribution of perineural invasion in the TCGA-PRAD dataset, which is mainly composed of a cohort of intermediate- to high-risk prostate cancer cases. Given that PNI is more frequently observed in these higher-risk groups, the predominance of the PNI group in our cohort is consistent with real-world clinical patterns and previous reports in the literature. Finally, survival analyses used in this study were univariate, as consistent data on PSA and stage were not available for all patients. Multivariable adjustment was not feasible without compromising sample size, particularly in the non-PNI group. Therefore, our prognostic findings should be considered hypothesis-generating and warrant validation in well-annotated, independent cohorts.

## 5. Conclusions

Our study demonstrates that PNI in prostate cancer is associated with distinct transcriptional, genetic, and pathway-level alterations. The identification of key hub genes and enriched biological processes provides a foundation for further investigations into the molecular mechanisms underlying PNI. Future research should focus on validating these findings in experimental models and assessing their clinical relevance, with the goal of developing targeted strategies to better manage PNI-associated disease.

## Data Availability

The original contributions presented in this study are included in this article. Further inquiries can be directed to the corresponding author.

## Abbreviations

PNI	perineural invasion
TCGA	the cancer genome atlas
NGS	next-generation sequencing
FFPE	formalin-fixed paraffin-embedded
RSEM	RNA-seq by expectation-maximization
DEGs	differentially expressed genes
PPI	protein–protein interaction
STRING	search tool for the retrieval of interacting genes
MCODE	molecular complex detection
GO	Gene Ontology
KEGG	Kyoto Encyclopedia of Genes and Genomes
WP	WikiPathways
OS	overall survival
DFS	disease-free survival
